# Relationship between atherosclerotic cardiovascular disease and diabetic retinopathy in patients with type 2 diabetes mellitus

**DOI:** 10.1097/MD.0000000000038051

**Published:** 2024-05-10

**Authors:** Li Li, Jiyun Gao, Xiaopang Rao, Xiaoling Liu

**Affiliations:** a Endocrinology and Metabolism Department, The Affiliated Hospital of Qingdao University, Qingdao, Shandong, China; b Department of Ophthalmology, Qingdao West Coast New Area District Hospital, Qingdao, Shandong, China; c Qingdao Chengyang District People’s Hospital, Qingdao, Shandong, China; d Pingdu City People Hospital, Qingdao, Shandong, China.

**Keywords:** atherosclerotic cardiovascular disease, correlation, diabetic retinopathy, proliferative diabetic retinopathy, type 2 diabetes mellitus

## Abstract

This study aimed to explore the potential correlation between atherosclerotic cardiovascular disease (ASCVD) and diabetic retinopathy (DR) in patients with type 2 diabetes mellitus (T2DM). We enrolled 6540 patients with T2DM who were receiving chronic disease management for hypertension, hyperglycemia, and hyperlipidemia in Chengyang District of Qingdao. Among them, 730 had ASCVD (ASCVD group), which 5810 did not (N-ASCVD group). The results showed significantly higher levels of age, blood glucose, glycosylated hemoglobin (HbA1c), systolic blood pressure, ASCVD family history, female proportion, and DR incidence in the N-ASCVD group. Additionally, the glomerular filtration rate was significantly lower in the ASCVD group. Logistic regression analysis revealed a positive correlation between DR and ASCVD risk. DR was further categorized into 2 subtypes, nonproliferative DR (NPDR) and proliferative DR (PDR), based on e lesion severity. Interestingly, only the PDR was associated with ASCVD. Even after accounting for traditional ASCVD risk factors such as age, sex, and family history, PDR remained associated with ASCVD, with a staggering 718% increase in the risk for patients with PDR. Therefore, there is a strong association between ASCVD and DR in individuals with T2DM, with PDR particularly exhibiting an independent and positive correlation with increased ASCVD risk.

## 1. Introduction

The prevalence of arteriosclerotic cardiovascular disease (ASCVD) is increasing rapidly, and it has become the main cause of death worldwide and among Chinese residents.^[1]^ A study showed that 70% of cardiovascular diseases (CVD) are caused by modifiable risk factors, including 41.2% metabolic factors (dyslipidemia, essential hypertension, diabetes, and obesity) and 26.3% behavioral risk factors (such as tobacco, alcohol, diet, physical activity, and sodium intake).^[2]^ Type 2 diabetes mellitus (T2DM) serves as a significant risk factor for ASCVD, which stands as the primary cause of mortality among individuals diagnosed with T2DM.^[3]^ In addition, patients in the early stages of ASCVD may exhibit asymptomatic or mild symptoms, potentially leading to oversight and delayed, ineffective treatment. Upon diagnosis, mortality and disability rates are notably elevated.^[3]^ Therefore, it is crucial to pay attention to the early screening and treatment of ASCVD.

Diabetic retinopathy (DR) is 1 of the most common microvascular complications of T2DM, and patients with DR have a higher risk of CVD and cardiovascular-related death.^[4–6]^ However, some studies suggest that patients with DR have an increased risk of CVD, which is related to traditional risk factors for ASCVD, such as blood glucose, blood lipids, and blood pressure. The value of DR in predicting cardiovascular events is low,^[7,8]^ and whether DR increases the risk of ASCVD remains unclear. This study analyzed patients with T2DM in chronic disease management in Chengyang District of Qingdao to elucidate the correlation between DR and ASCVD.

## 2. Methods

### 2.1. Study population

From September 2019 to December 2020, 6540 patients with T2DM were enrolled in the chronic disease management population with hypertension, hyperglycemia, and hyperlipidemia in Chengyang District of Qingdao, including 730 patients with ASCVD (ASCVD group) and 5810 patients without ASCVD (N-ASCVD group). The resident was of Chinese nationality and had lived in the area for at least 6 months. The diagnosis of T2DM conforms to the diagnostic criteria of the 2020 Chinese guidelines for the prevention and treatment of T2DM. The exclusion criteria were as follows: incomplete case data; presence of other endocrine diseases; chronic obstructive pulmonary disease; rheumatic immune diseases; Glaucoma, Cataract, Ocular trauma, and other diseases affecting fundus grading; acute infection; malignant tumor; Pregnancy; Type 1 diabetes mellitus; secondary diabetes mellitus; chronic pancreatitis; and estimated glomerular filtration rate (eGFR) <60 mL/minutes. ASCVD is diagnosed with CVD, including acute coronary syndrome, myocardial infarction, angina pectoris, coronary or other revascularization, atherogenic stroke, transient ischemic attack, and atherogenic peripheral artery disease. This study was approved by the Medical Ethics Committee of the Qingdao Chengyang District People’s Hospital (CYQRMYY2019-0623).

### 2.2. Basic data

Basic information included the following: name, sex, date of birth, household registration, education level, marital status, occupation, smoking, drinking, diet, physical activity, and family history of chronic diseases.

### 2.3. Anthropometric indicators

Height, body weight, waist circumference, and blood pressure were measured thrice to obtain the mean. Body mass index (BMI) = body weight/height 2 (kg/m^2^).

### 2.4. Biochemical indexes

The subjects were required to fast for 10 hours and blood samples were collected. Blood glucose, serum creatinine, total cholesterol, triglycerides, high-density lipoprotein cholesterol, and low-density lipoprotein cholesterol (LDL-C) were measured according to previous studies.^[9,10]^ Glycosylated hemoglobin (HbA1c) was measured using a high-pressure liquid phase (VARIANT ii Analyzer, Bio-Rad). The oral glucose tolerance test and eGFR were also performed using an automatic biochemical detector (Roche).

### 2.5. Fundus examination and evaluation

All patients were evaluated by 2 ophthalmologists from the Chengyang District People’s Hospital of Qingdao according to the 2002 International Clinical Classification of DR^[11]^: no obvious retinopathy, nonproliferative diabetic retinopathy (NPDR), Moderate NPDR, Severe NPDR, and proliferative DR (PDR). The ophthalmologist performed mydriasis and slit-lamp examinations, independently interpreting the films simultaneously. Staging was determined upon consensus. In cases where consensus was not achieved, senior doctors were consulted for film interpretation and staging.

### 2.6. Disease diagnostic criteria

Hypertension^[12]^: Patients with previously diagnosed hypertension and taking antihypertensive drugs; Blood pressure measurement systolic blood pressure ≥ 140 mm Hg and/or diastolic blood pressure ≥ 90 mm Hg. Overweight and obesity^[13]^: 28 kg/m^2^ > BMI ≥ 24 kg/m^2^ was considered overweight and BMI ≥ 28 kg/m^2^ was considered obese. Abdominal obesity^[14]^: waist circumference ≥ 90 cm for men and ≥ 80 cm for women. Smoking was defined as ≥ 1 cigarette per day for more than 1 year. The average monthly drinking duration in the past year was ≥ 1 time. Physical exercise: Moderate or vigorous activity for 30 minutes per day for at least 3 days per week. High-level education: College degree or above.

### 2.7. Statistical analysis

The SPSS software (version 23.0) was used to establish a database for statistical analysis. An independent sample t test was used for comparisons between the 2 groups. The median was used for measurement data with nonnormal distribution, and the Mann–Whitney U test was used for comparison between groups. The qualitative data rate (%) was expressed as a *χ*^2^ test for comparison between groups. A multivariate logistic regression, *P* < .05, was considered statistically significant.

## 3. Results

### 3.1. Clinical data analysis of patients

A total of 6540 patients with an average age of 53.1 ± 10.1 years, included 2950 males and 3590 females. In comparison to the N-ASCVD group, the ASCVD group exhibited significantly higher mean age, PG 2 hours, HbA1c levels, blood pressure, ASCVD family history, and female proportion. The proportion of smokers and drinkers and eGFR levels were significantly lower in the ASCVD group compared to the N-ASCVD group (*P* < .05) (Table 1).

**Table 1:** General information and biochemical test results of the study objects.

	Total	Non-ASCVD	ASCVD	t/Z/x^2^	*P* value
Number (%)	6540 (100)	5810 (88.8)	730 (11.2)		
Age (yr)^a^	53.20 (47.0, 59.2)	52.40 (46.5, 58.3)	58.50 (53.9, 65.9)	−5.86^e^	.00
Female, *n* (%)	3590 (54.80)	3070 (52.80)	520 (71.20)	−8.86	.00
Urban registration, *n* (%)	2160 (33.0)	1930 (33.20)	230 (31.50)	0.09	.77
Smoking, *n* (%)	1990 (30.40)	1880 (32.40)	110 (15.1)	9.16	.00
Alcohol, *n* (%)	2210 (33.70)	2080 (35.80)	130 (17.80)	9.38	.00
High educational level, *n* (%)	760 (11.60)	710 (12.20)	50 (6.90)	1.75	.19
Physical activity, *n* (%)	4810 (73.50)	4240 (73.00)	570 (78.10)	0.87	.35
Family history of ASCVD, *n* (%)	3010 (46.00)	2560 (44.10)	450 (61.60)	8.07	.01
Waistline, *n* (%)	90.00 ± 10.00	89.80 ± 10.00	91.20 ± 9.90	−1.07^d^	.28
Central obesity, *n* (%)	4070 (62.20)	3540 (60.90)	530 (72.60)	3.76	.05
BMI (kg/m^2^)^a^	26.60 (24.40, 28.90)	26.70 (24.40, 28.90)	26.27 (24.12, 29.01)	−0.44^e^	.66
General obesity, *n* (%)	5100 (77.90)	4540 (78.10)	560 (76.70)	0.08	.78
Duration of DM (yr)^b^	10.50 ± 6.70	10.55 ± 6.60	10.20 ± 7.20	0.41^d^	.68
FPG (mmol/L)^a^	7.83 (6.77, 9.44)	7.77 (6.77, 9.38)	8.38 (6.96, 10.65)	−1.76^e^	.08
PG 2 h (mmol/L)^a^	13.21 (9.10, 18.13)	13.10 (8.99, 17.93)	16.26 (11.08, 19.20)	−2.35^e^	.02
HbAlc (%)^a^	6.80 (6.00, 7.90)	6.70 (6.00, 7.90)	7.20 (6.55, 8.85)	−3.33^e^	.00
Hypertension, *n* (%)	4690 (71.70)	4110 (70.70)	580 (79.50)	2.43	.12
SBP (mm Hg)^a^	143 (131, 159)	143 (131, 158)	151 (133, 165)	−2.28^e^	.02
DBP (mm Hg)^a^	87 (79, 94)	87 (80, 95)	87 (78, 93)	−0.78^e^	.44
TC (mmol/L) ^a^	5.22 (4.58, 5.98)	5.21 (4.58, 5.93)	5.26 (4.45, 6.22)	−0.94^e^	.35
TG (mmml/L)^b^	1.51 (1.01, 2.23)	1.44 (1.05, 2.08)	1.61 (1.10, 2.30)	0.99^c^	.32
LDL-C (mmol/L) ^b^	2.67 ± 0.53	2.66 ± 0.52	2.72 ± 0.62	−0.89^d^	.37
HDL-C (mmol/L) ^b^	1.42 ± 0.29	1.42 ± 0.29	1.46 ± 0.33	−1.23^c^	.22
eGFR (mL**/**min^−1^**/**1.73 m^−2^)^a^	93.2 (84.1, 106.1)	94.6 (84.8, 106.3)	87.2 (75.0, 103.0)	−3.47^e^	.00

ASCVD = atherosclerotic cardiovascular disease, BMI = body mass index, eGFR = glomerular filtration rate. A data M (Q1, Q3); B data ± s; C is Z value; D t value, FPG = fasting blood glucose, HbAlc = glycosylated hemoglobin, HDL-C = high-density lipoprotein cholesterol, LDL-C = low-density lipoprotein cholesterol, PG 2 h = after taking sugar 2 h blood glucose, TG = triglycerides.

### 3.2. Comparison of DR and different degrees of DR between the 2 groups

Among the 730 subjects with ASCVD, 140 (19.2%) exhibited DR, while 590 (80.8%) did not. In the N-ASCVD group, 480 (8.3%) had DR, while 5330 (91.7%) did not; the difference was statistically significant (*χ*^2^ = 9.01, *P* < .001). Furthermore, DR was divided into NPDR and PDR, based on the degree of severity. In the ASCVD group, there were 100 cases (13.7%) with NPDR and 40 cases (5.5%) with PDR, which were higher than the 450 cases (7.7%) (*χ*^2^ = 3.57, *P* = .06) and 30 cases (0.5%) (*χ*^2^ = 16.33, *P* < .001) in the N-ASCVD group (Table 1).

### 3.3. The effects of DR and different degrees of DR on ASCVD

Logistic regression analysis showed that DR positively correlated with ASCVD. Only PDR was related to ASCVD. After adjusting for age, sex, household registration, family history of ASCVD, duration of diabetes (Model 1), smoking, drinking, education level, physical exercise, obesity, hypertension, systolic blood pressure, diastolic blood pressure, HbA1c, LDL-C, and eGFR (Model 2), PDR was still associated with ASCVD, with a 718% increased risk of ASCVD in patients with PDR (Table 2).

**Table 2:** Logistic regression analysis results of the influence of DR on ASCVD.

	B	SE	*χ* ^2^	95%CI	*P* value
DR					
ASCVD	0.97	0.33	8.45	2.64 (1.37–5.06)	.00
Model 1	0.96	0.36	7.18	2.634 (1.31–5.30)	.00
Model 2	0.70	0.41	2.89	2.107 (0.93–4.78)	.07
NPDR					
ASCVD	0.70	0.38	3.44	2.01 (0.96–4.19)	.06
Model 1	0.67	0.40	2.79	1.98 (0.99–4.35)	.09
Model 2	0.42	0.47	0.80	1.52 (0.61–3.78)	.37
PDR					
ASCVD	2.49	0.78	10.29	12.05 (2.63–55.12)	.00
Model 1	2.34	0.81	8.40	10.69 (2.20–51.87)	.00
Model 2	2.10	0.84	6.20	8.18 (1.56–42.81)	.01

ASCVD = atherosclerotic cardiovascular disease, DR = diabetic retinopathy, NPDR = nonproliferative DR, PDR = proliferative phase DR; Model 1 was adjusted by age, gender, household registration, family history of ASCVD and duration of diabetes. Model 2 was further adjusted for smoking history, drinking history, education level, physical exercise, obesity (abdominal type, systemic type), history of hypertension, systolic blood pressure, diastolic blood pressure, HbA1c, LDL-C, and eGFR on model 1.

## 4. Discussion

ASCVD refers to acute or chronic ischemic lesions in multiple vascular bed-dominated areas of the body.^[15]^ Six risk factors, namely age, hypertension, smoking, plasma total cholesterol level, plasma high-density lipoprotein level, and diabetes, were used to assess ASCVD. Therefore, clinical risk factors have become the primary criteria for the international cardiovascular community to assess the degree of ASCVD risk in asymptomatic individuals and to guide primary prevention measures. In this study, the mean age, PG 2 hours, HbA1c, systolic blood pressure, family history of ASCVD, and proportion of female patients in the ASCVD group were significantly higher than those in the non-ASCVD group, which is similar to previous findings.^[16]^ Smoking and drinking are the traditional risk factors for ASCVD. However, the prevalence of smoking and drinking was significantly lower in the ASCVD group than in the non-ASCVD group. This result was related to the fact that the prevalence of smoking (5.6%) and drinking (7.5%) in women was significantly lower than in men (60.7%) and drinking (65.8%). The proportion of women in the ASCVD group was significantly higher than that of men (71.2% vs 28.8%), while the proportion of women and men in the non-ASCVD group (52.8% vs 47.2%) was not significantly different, indicating that there was a certain relationship between the sex ratio difference between the 2 groups.

Microvascular and macrovascular lesions in T2DM share a common pathophysiological basis. Previous studies have shown that obesity, hypertension, hyperglycemia, and metabolic syndrome are closely related to DR lesions and that these are also related risk factors for ASCVD; therefore, DR and ASCVD have similar risk factors.^[17,18]^ Microvascular dysfunction and mechanisms of blood vessel damage share common pathways, including the hexose amine pathway, protein kinase C pathway, oxidative stress injury, renin–angiotensin–aldosterone system, chronic inflammation, growth factors, and blood rheology changes.^[19,20]^ These mechanisms not only affect the occurrence and development of DR but also the progression of atherosclerosis.^[21]^

In terms of epidemiology, the action to control cardiovascular risk in patients with diabetes (ACCORD) study of 3433 diabetic patients with an average age of 61 years showed that compared to subjects without DR, the relative risks of cardiovascular events (cardiovascular death or nonfatal myocardial infarction or stroke) in patients with mild and severe DR were 1.49 (95%CI: 1.12–1.97) and 2.35 (95%CI: 1.47–3.76), respectively.^[6]^ This study showed that DR was significantly correlated with the occurrence of ASCVD and that the risk of ASCVD increased the severity of DR lesions. Moreover, the PDR is an independent risk factor for ASCVD. The presence of PDR significantly increased the risk of ASCVD in patients by 7.18 times. Patients with PDR have a higher risk of ASCVD than patients with NPDR, which may be due to the longer course of diabetes, greater burden of atherosclerosis, and more severe insulin resistance.^[22]^

The 4-year follow-up of 2856 patients in the ACCORD study showed that the risk of the primary outcome increased by 38% (1.38 [95%CI: 1.10–1.74]) as the severity of DR increased.^[7]^ Kurtul et al^[23]^ investigated 96 patients with T2DM who underwent coronary angiography. Fundus examination was conducted to evaluate the presence and severity of Dr The SYNTAX score was calculated for each patient to assess the severity of coronary artery disease. Results indicated a significant correlation between SYNTAX score and the extent of coronary artery disease in T2DM patients, with a higher SYNTAX score (>16.5) emerging as a crucial independent predictor of DR.^[23]^ Another study of a population without ASCVD showed that PDR could predict all-cause ASCVD mortality compared to individuals without DR after adjusting for ASCVD-related risk factors.^[8]^ Our results demonstrated the independent and positive correlation between PDR and ASCVD risk.

This study has indicated that this cross-sectional study may have unpredictable bias. Second, retrospective case–control studies cannot directly assess the causal relationship between DR and ASCVD. Therefore, it is necessary to further expand the sample size and conduct long-term prospective cohort studies to observe the occurrence of ASCVD and understand the relationship between DR and ASCVD.

## Author contributions

**Conceptualization:** Xiaoling Liu.

**Data curation:** Li Li, Xiaopang Rao, Xiaoling Liu.

**Funding acquisition:** Li Li.

**Formal analysis:** Jiyun Gao, Xiaoling Liu.

**Investigation:** Xiaopang Rao.

**Methodology:** Xiaopang Rao.

**Resources:** Jiyun Gao.

**Software:** Jiyun Gao.

**Supervision:** Xiaopang Rao.

**Validation:** Li Li, Jiyun Gao, Xiaopang Rao, Xiaoling Liu.

**Writing – original draft:** Li Li.

**Writing – review & editing:** Xiaoling Liu.

## References

[1] Xie J Ikram MK Cotch MF. Association of diabetic macular edema and proliferative diabetic retinopathy with cardiovascular disease: a systematic review and meta-analysis. JAMA Ophthalmol. 2017;135:586–93. 28472362 10.1001/jamaophthalmol.2017.0988 PMC5593137

[2] Yusuf S Joseph P Rangarajan S. Modifiable risk factors, cardiovascular disease, and mortality in 155 722 individuals from 21 high-income, middle-income, and low-income countries (PURE): a prospective cohort study. Lancet. 2020;395:795–808. 31492503 10.1016/S0140-6736(19)32008-2 PMC8006904

[3] O’Keefe JH Carter MD Lavie CJ. Primary and secondary prevention of cardiovascular diseases: a practical evidence-based approach. Mayo Clin Proc. 2009;84:741–57. 19648392 10.4065/84.8.741 PMC2719528

[4] Guo VY Cao B Wu X. Prospective association between diabetic retinopathy and cardiovascular disease – a systematic review and meta-analysis of cohort studies. J Stroke Cerebrovasc Dis. 2016;25:1688–95. 27068777 10.1016/j.jstrokecerebrovasdis.2016.03.009

[5] Mottl AK Pajewski N Fonseca V. The degree of retinopathy is equally predictive for renal and macrovascular outcomes in the ACCORD trial. J Diabetes Complications. 2014;28:874–9. 25123755 10.1016/j.jdiacomp.2014.07.001 PMC4252726

[6] Gerstein HC Ambrosius WT Danis R. Diabetic retinopathy, its progression, and incident cardiovascular events in the ACCORD trial. Diabetes Care. 2013;36:1266–71. 23238658 10.2337/dc12-1311 PMC3631868

[7] Juutilainen A Lehto S Ronnemaa T. Retinopathy predicts cardiovascular mortality in type 2 diabetic men and women. Diabetes Care. 2007;30:292–9. 17259497 10.2337/dc06-1747

[8] Targher G Bertolini L Tessari R. Retinopathy predicts future cardiovascular events among type 2 diabetic patients: the Valpolicella Heart Diabetes Study. Diabetes Care. 2006;29:1178. 10.2337/diacare.2951178 16644664

[9] Dang Y Xu J Yang Y. Ling-gui-zhu-gan decoction alleviates hepatic steatosis through SOCS2 modification by N6-methyladenosine. Biomed Pharmacother. 2020;127:109976. 32559839 10.1016/j.biopha.2020.109976

[10] Dang Y Xu J Zhu M. Gan-Jiang-Ling-Zhu decoction alleviates hepatic steatosis in rats by the miR-138-5p/CPT1B axis. Biomed Pharmacother. 2020;127:110127. 32325349 10.1016/j.biopha.2020.110127

[11] Wilkinson CP Ferris FL 3rd Klein RE. Proposed international clinical diabetic retinopathy and diabetic macular edema disease severity scales. Ophthalmology. 2003;110:1677–82. 13129861 10.1016/S0161-6420(03)00475-5

[12] James PA Oparil S Carter BL. 2014 evidence-based guideline for the management of high blood pressure in adults: report from the panel members appointed to the Eighth Joint National Committee (JNC 8). JAMA. 2014;311:507–20. 24352797 10.1001/jama.2013.284427

[13] Loayza D Carpousis AJ Krisch HM. Gene 32 transcription and mRNA processing in T4-related bacteriophages. Mol Microbiol. 1991;5:715–25. 2046553 10.1111/j.1365-2958.1991.tb00742.x

[14] Fang H Berg E Cheng X. How to best assess abdominal obesity. Curr Opin Clin Nutr Metab Care. 2018;21:360–5. 29916924 10.1097/MCO.0000000000000485 PMC6299450

[15] Libby P Buring JE Badimon L. Atherosclerosis. Nat Rev Dis Primers. 2019;5:56. 31420554 10.1038/s41572-019-0106-z

[16] Gao L Zhao W Yang JK. Proliferative diabetic retinopathy in patients with type 2 diabetes correlates with the presence of atherosclerosis cardiovascular disease. Diabetol Metab Syndr. 2021;13:48. 33902673 10.1186/s13098-021-00666-z PMC8077820

[17] Osborn EA Jaffer FA. Imaging inflammation and neovascularization in atherosclerosis: clinical and translational molecular and structural imaging targets. Curr Opin Cardiol. 2015;30:671–80. 26398413 10.1097/HCO.0000000000000226 PMC4632070

[18] Simo R Carrasco E Garcia-Ramirez M. Angiogenic and antiangiogenic factors in proliferative diabetic retinopathy. Curr Diabetes Rev. 2006;2:71–98. 18220619 10.2174/157339906775473671

[19] Zhang C Wang H Nie J. Protective factors in diabetic retinopathy: focus on blood-retinal barrier. Discov Med. 2014;18:105–12. 25227751

[20] Martin-Timon I Sevillano-Collantes C Segura-Galindo A. Type 2 diabetes and cardiovascular disease: have all risk factors the same strength? World J Diabetes. 2014;5:444–70. 25126392 10.4239/wjd.v5.i4.444 PMC4127581

[21] Ellis TP Choudhury RH Kaul K. Diabetic retinopathy and atherosclerosis: is there a link? Curr Diabetes Rev. 2013;9:146–60. 23094754 10.2174/1573399811309020006

[22] Gimeno-Orna JA Faure-Nogueras E Castro-Alonso FJ. Ability of retinopathy to predict cardiovascular disease in patients with type 2 diabetes mellitus. Am J Cardiol. 2009;103:1364–7. 19427429 10.1016/j.amjcard.2009.01.345

[23] Kurtul BE Kurtul A Yalcin F. Predictive value of the SYNTAX score for diabetic retinopathy in stable coronary artery disease patients with a concomitant type 2 diabetes mellitus. Diabetes Res Clin Pract. 2021;177:108875. 34058301 10.1016/j.diabres.2021.108875

